# Rapid Eye Movement Sleep Behavior Disorder and Neurodegenerative Diseases: An Update

**DOI:** 10.14336/AD.2019.0324

**Published:** 2020-03-09

**Authors:** Feng Zhang, Long Niu, Xinyao Liu, Yufei Liu, Song Li, Huan Yu, Weidong Le

**Affiliations:** ^1^Center for Clinical Research on Neurological Diseases, the First Affiliated Hospital, Dalian Medical University, Dalian, China.; ^2^Liaoning Provincial Key Laboratory for Research on the Pathogenic Mechanisms of Neurological Diseases, the First Affiliated Hospital, Dalian Medical University, Dalian, China.; ^3^Sleep and Wake Disorders Center and Department of Neurology, Huashan Hospital, Fudan University, Shanghai, China.

**Keywords:** rapid eye movement sleep behavior disorder, synucleinopathy, dementia, neurodegeneration

## Abstract

Rapid eye movement sleep behavior disorder (RBD) is a sleep behavior disorder characterized by abnormal behaviors and loss of muscle atonia during rapid eye movement (REM) sleep. RBD is generally considered to be associated with synucleinopathies, such as Parkinson’s disease (PD), dementia with Lewy bodies (DLB), and multiple system atrophy (MSA), and usually precedes years before the first symptom of these diseases. It is believed that RBD predicts the neurodegeneration in synucleinopathy. However, increasing evidences have shown that RBD is also found in non-synucleinopathy neurodegenerative diseases, including Alzheimer’s disease (AD), Huntington’s disease (HD), amyotrophic lateral sclerosis (ALS), etc. Sleep disturbance such as RBD may be an early sign of neurodegeneration in these diseases, and also serve as an assessment of cognitive impairments. In this review, we updated the clinical characteristics, diagnosis, and possible mechanisms of RBD in neurogenerative diseases. A better understanding of RBD in these neurogenerative diseases will provide biomarkers and novel therapeutics for the early diagnosis and treatment of the diseases.

Rapid eye movement sleep behavior disorder (RBD) is a parasomnia characterized by abnormal behaviors and loss of muscle atonia, such as vocalizations, jerks and motor behaviors during rapid eye movement (REM) sleep, often related to REM-related dream content [1]. The prevalence of RBD is estimated to be 0.5-2%, but larger population-based studies of probable dream enactment symptoms suggest RBD is likely more frequent (5-13%) in older community-dwelling adults [2]. The incidence of RBD is approximately equal in men and women under the age of 50, but it appears to be more common in men than women in older adults [3]. Maybe it is because women have less violent dream enactment behaviors (DEBs) [4]. RBD can be categorized into idiopathic or symptomatic forms [3]. Idiopathic RBD (iRBD) often occurs in the absence of ongoing neurological conditions, whereas symptomatic RBD is related to identifiable underlying etiologies [5], such as α-synuclein (α-syn) pathology and other forms of neurodegeneration, pontine lesions, and toxic effects from medications. Some evidence suggests that both idiopathic and symptomatic forms of RBD are associated with neurodegenerative diseases, especially α-syn related diseases [4]. iRBD is considered to be an important precursor of synucleinopathies such as Parkinson's disease (PD), dementia with Lewy bodies (DLB) and multiple system atrophy (MSA) [6, 7]. In fact, up to 82% of older men diagnosed with RBD develop parkinsonism or dementia [8]. Almost half of the PD patients, at least 88% of the MSA patients, and about 80% of the DLB patients have RBD [3]. Study has reported that up to 98% of individuals with polysomnography (PSG)-confirmed RBD have synucleinopathy [9]. Symptomatic RBD may also be associated with narcolepsy, Guillain Barre syndrome, limbic encephalitis and Morvan’s syndrome [10]. RBD is recently recognized as part of the clinical spectrum of IgLON5-anti-associated sleep disorder [11]. Besides α-syn diseases, RBD has been reported in non-synucleinopathies, such as Alzheimer’s disease (AD) [12], progressive supranuclear palsy (PSP) [13], Huntington’s disease (HD) [14], and amyotrophic lateral sclerosis (ALS) [15].

## Clinical features and diagnosis of RBD

RBD can cause sleep disruption, excessive motor activity ranging from simple limb twitches to violent, complex movements that may cause injury to the patients and/or sleeping partners [5]. These behaviors are often referred to as DEBs because patients often recall dreams immediately after waking up following an episode, and these behaviors seem to be purposeful [16]. Patients with RBD often have violent dream content [17]. The behaviors can occur more towards the morning hours because that is when REM sleep is more frequent, and the behaviors can start as early as 90 minutes after the first REM sleep episode [16]. Recent studies have showed that Epworth sleep scale > 8 means more rapid conversion to PD and dementia at the time of iRBD diagnosis, and in some iRBD patients, cognitive impairment may reflect early stages of neurodegenerative diseases [18]. And if the patients with iRBD have hyposmia and constipation, orthostatic hypotension, and gait abnormalities, suggesting possible underlying pathologies of synucleinopathy [2].

The 3^rd^ revision diagnostic criteria of International Classification of Sleep Disorders Criteria for REM sleep behavior disorders consists (1) repeated sleep-related vocalizations and/or complex motor behaviors; (2) these behaviors are documented by polysomnography to occur during REM sleep, or based on the clinical history of dream enactment, are presumed to occur during REM sleep; (3) polysomnographic demonstrates REM sleep without atonia (RSWA); (4) the sleep disturbance is not better explained by another sleep disorder, mental disorder, medication, or substance use. [19].

Diagnosis of RBD requires either a clinical history of DEBs or REM sleep-related behaviors recorded by PSG, along with RSWA [2]. The witness of dream enactment by bed partner is the key to diagnose RBD [20]. RSWA is the electrophysiological hallmark of RBD, and also a key diagnostic feature of RBD [10, 21]. Some questionnaires can also be used in the diagnosis of RBD [22], such as RBD Screening Questionnaire (RBDSQ), the REM Sleep Behavior Questionnaires-Hong-Kong (RBD-HK), the Mayo Sleep Questionnaire (MSQ) and the Innsbruck RBD Inventory [2]. RBD needs to be distinguished from other similar diseases, such as non-REM parasomnias (sleepwalking, sleep terrors), periodic limb movement disorder, severe obstructive sleep apnea, and dissociative disorders, therefore PSG is very important for the diagnosis of RBD [10, 21]. Neuroimaging techniques might be helpful in detecting structural changes in RBD patients’ brains. For example, diffusion tensor imaging (DTI), a widely used magnetic resonance imaging (MRI) technique, has been reported to detect white matter abnormalities in patients with iRBD [23]. White matter changes have been found in the brainstem where REM is modulated, and other brain areas, such as the right substantia nigra, the olfactory region, the left temporal lobe, the fornix, the internal capsule, the corona radiata, and the right visual stream in the patients with iRBD [23, 24]. However, other DTI studies have revealed slight or moderate changes in white matter of iRBD patients [25, 26]. A study of 3.0-T MRI has showed the loss of nigral hyperintensity in 11 iRBD patients, in consistent with a significant lower ^123^I-N-3-fluoropropyl-2β-carbomethoxy -3β-4-iodophenyl tropane (a visualized tracer of striatal pre-synaptic dopamine transporter) uptake ratios [27]. Moreover, in a follow-up study of 5 iRBD patients with nigral hyperintensity loss, all patients have developed symptoms of parkinsonism or dementia 18 months after neuroimaging, implying that nigral hyperintensity loss at 3.0-T susceptibility-weighted imaging might be a marker for synucleinopathy risk in iRBD [27]. Overall, definite diagnosis of RBD is based on PSG confirmation. For identification or screening in large populations, single screening questions could be used followed by specific RBD rating scales and a more detailed sleep interview [28]. Other clinical symptoms such as olfactory loss, autonomic dysfunction, cognitive impairment, and examinations such as functional MRI, transcranial sonography, and peripheral nerve tissue biopsies may be helpful for mornitoring the outcome of RBD [28].

### Pathogenic mechanisms of RBD

RBD may be triggered by neurodegenerative diseases or associated with antidepressant treatments, alcohol, and drug withdrawal [4]. But the mechanisms underlying the pathogenesis of RBD are still not clear. In rodents, the core of the REM-generating circuit includes γ-aminobutyric acid (GABA)-ergic neurons located in the lateral hypothalamus, dorsal paragigantocellular reticular nucleus, and ventrolateral periaqueductal grey (vlPAG) which inactivates REM-inhibiting monoaminergic neurons in the tuberomammillary nucleus, locus coeruleus (LC), and dorsal raphe and GABA-ergic neurons in the vlPAG to induce REM sleep [29]. The pre-coeruleus and sublaterodorsal nucleus (SLD) (equivalent to subcoeruleus nucleus (SubCD) in humans [30]) induce atonia during REM sleep in rodents [2]. SLD glutamatergic/GABA-ergic neurons stimulate inhibitory spinal interneurons, which in turn inhibit or hyperpolarize motor neurons to produce skeletal muscle atonia or stimulates glycinergic and GABA-ergic premotor neurons in the ventromedial medulla (VMM), including raphe magnus and lateral gigantocellular reticular nuclei (ventral gigantocellular, alpha gigantocellular and lateral paragigantocellular reticular nuclei), which result in inhibition of motor neurons and cause skeletal muscle atonia [2, 16, 31]. Blocking glycine and GABA receptors in the VMM via both pharmacological and genetic ways increases muscle twitches during REM sleep [29]. The cortical activation during REM sleep is restricted to a few limbic structures, such as retrosplenial cortex, medial entorhinal cortex, anterior cingulate cortex and the dentate gyrus, which produce dream and activate the motor cortex, leading to the activation of spinal motor neurons [31].

**Figure 1.**
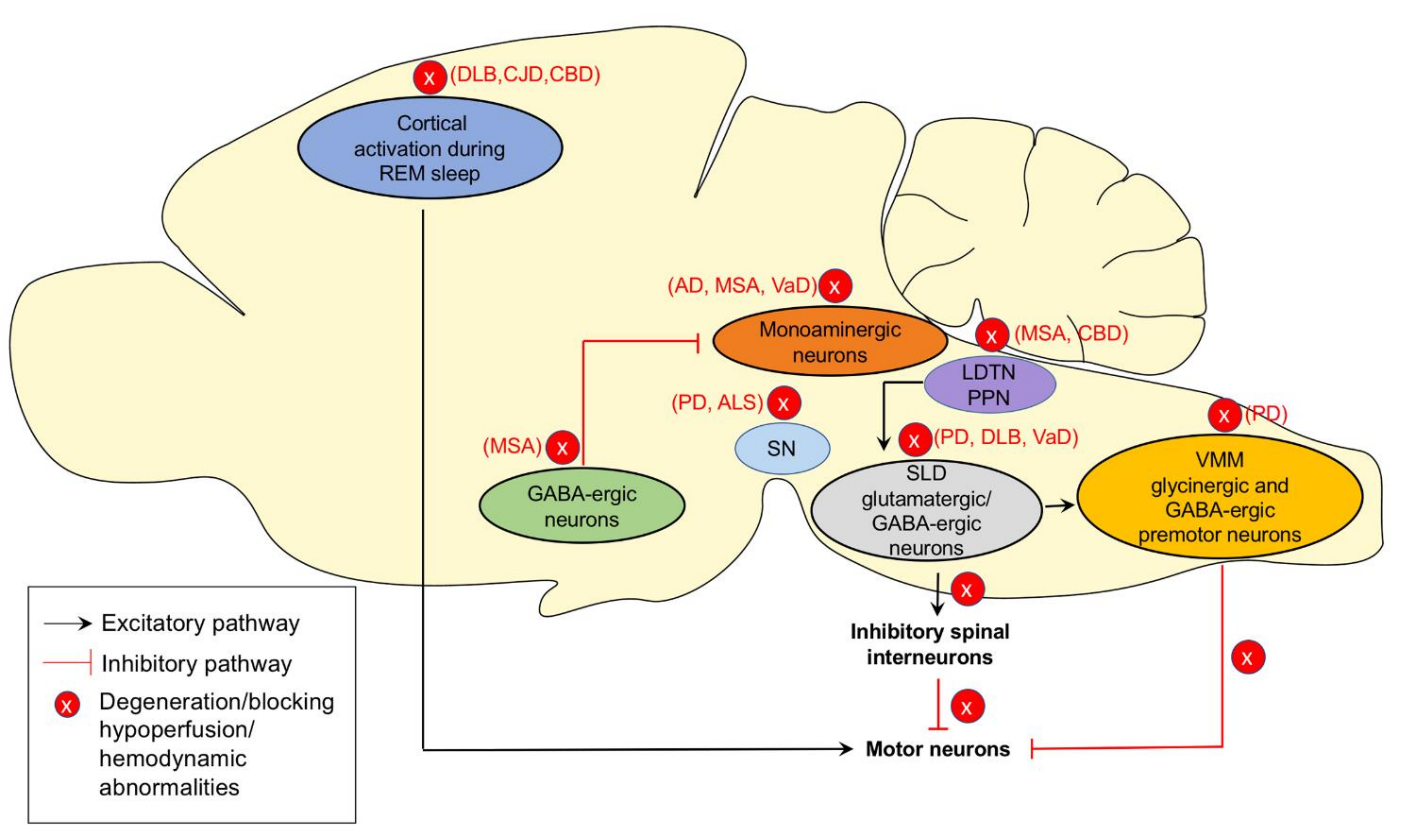
The potential mechanisms of RBD and related pathological pathways associated with neurodegenerative diseases. During rapid eye movement (REM) sleep, the γ-aminobutyric acid (GABA)-ergic neurons located in the lateral hypothalamus and other nuclei inactivate REM-inhibiting monoaminergic neurons in the tuberomammillary nucleus, locus coeruleus (LC), and dorsal raphe and GABA-ergic neurons in the ventrolateral periaqueductal grey (vlPAG) to induce REM sleep. Sublaterodorsal nucleus (SLD) glutamatergic/GABA-ergic neurons stimulate inhibitory spinal interneurons or glycinergic and GABA-ergic premotor neurons in the ventromedial medulla (VMM) resulting in skeletal muscle atonia. SLD neurons may be activated by the cholinergic laterodorsal tegmental nucleus and pedunculopontine tegmental nucleus (LDTN/PPN) neurons. The cortical activation during REM sleep leads to the activation of spinal motor neurons. Blocking glycine and GABA receptors or the degeneration of glycinergic and GABA-ergic neurons in the SLD and VMM removes the inhibition of spinal motor neurons and prevents the induction of muscle atonia. This could be the possible mechanism of RBD. In neurodegenerative diseases, pathological changes affecting the REM sleep regulating nuclei and circuits may contribute to the pathogenesis of RBD in these specific diseases.

In healthy individuals, motor neuronal activation is inhibited by the SubCD and VMM, but in patients with RBD, degeneration of glycinergic and GABA-ergic neurons in the SubCD and VMM removes the inhibition of spinal motor neurons and prevents the induction of muscle atonia [31]. Many other sites are also involved in REM sleep regulation, including pedunculopontine nucleus, laterodorsal tegmental nucleus (LDTN), thalamus, substantia nigra, basal forebrain, and frontal cortex [4]. The posterior hypothalamic hypocretin may have the effect of stabilizing REM-generating and REM-inhibiting centers and networks [32]. The red nucleus, pedunculopontine nucleus, and LDTN may be the source of muscle twitches during REM sleep [32]. The circuits that regulate REM sleep in the caudal brainstem are the same structures where α-syn pathology might begin [30]. Lesions in these structures are thought to eliminate atonia during REM sleep [4, 33]. Autopsy evidence implies that RBD patients have degeneration in brainstem nuclei that control REM sleep, with Lewy bodies and Lewy neurites in these areas [34]. Hypothesis suggests that lesions in the REM sleep circuits can lead to RBD-like motor behavior in both animals (cats, rats, mice) and humans [35]. Braak has used postmortem analysis of PD brains to propose a hypothesis that the α-syn pathology progressively develops in a caudal to rostral fashion, this progression might be caused by cell-to-cell transmission between interconnected brain regions [36, 37]. The pathological changes begin from medulla and pons, which may be related to the development of initial RSWA and RBD in idiopathic RBD and eventually ascending to more rostral structures [2, 36]. In most cases iRBD may not be separate disease entity from synucleinopathies, but rather an early manifestation of synaptophysin diseases.

**Figure 2.**
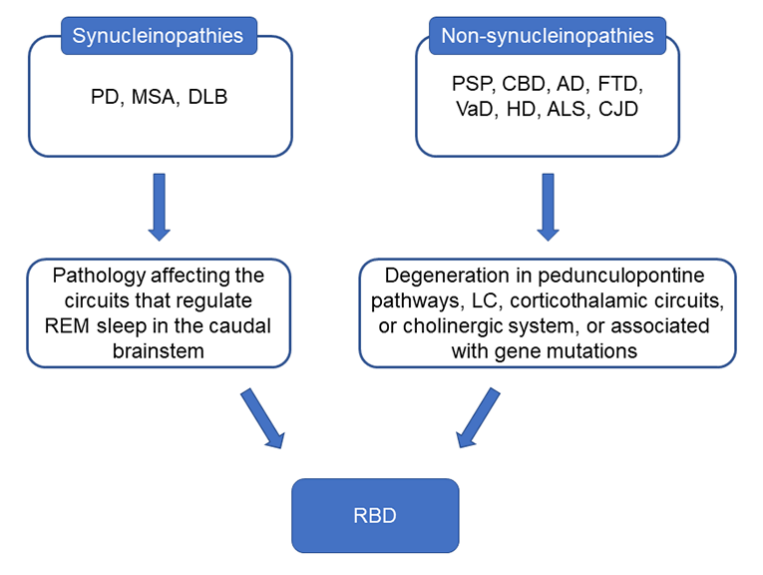
The possible mechanisms of RBD associated with synucleinopathies or non-synucleinopathies. Lesions in caudal brainstem are thought to eliminate atonia during REM sleep and the brainstem is the same structure where α-syn pathology might begin in synucleinopathies. In non-synucleinopatheis, the pathological changes may affect pedunculopontine pathways, locus coeruleus (LC), corticothalamic circuits or cholinergic system and finally affect the REM sleep regulating systems to induce RBD.

In non-synucleinopathies, pathological changes affect pedunculopontine tegmentum or cholinergic system may induce RBD. For example, loss of cholinergic neurons in the pedunculopontine tegmentum in PSP and degenerative process in the nuclei of the brainstem and pedunculopontine pathways in corticobasal degeneration (CBD) may cause RBD in these patients [13, 38]. Moreover, in frontotemporal dementia (FTD) and ALS where α-syn pathology is rarely found, RBD might exist alone without α-syn. Degeneration of neurons in REM-associated pathways in the brainstem might contribute to RBD in ALS [15]. The plausible underlying mechanisms of RBD and related pathological pathways associated with neurodegenerative diseases are represented in Figure 1 and Figure 2.

So far, the research on the genetic factors of RBD is limited. Study shows that in patients with idiopathic RBD and PD, there is a close association between RBD and the glucose encephalo-glucosidase (*GBA*) mutation [39]. PD-associated loci, scavenger receptor class B member 2 (*SCARB2*) rs6812193 and microtubule associated protein tau (*MAPT*) rs12185268 are also associated with RBD, while homozygous carriers of the ubiquitin specific peptidase 25 (*USP25*) rs2823357 single-nucleotide polymorphism (SNP) show a more rapid progression to synucleinopathy [40]. In RBD patients, PTEN-induced putative kinase 1 (*PINK1*) SNP rs45478900 carriers could have a higher risk of conversion to PD [41]. The leucine rich repeat kinase 2 (*LRRK2*) mutation have some connection with RBD and PD [2].

### RBD and Parkinson’s disease

PD is a common and complex neurodegenerative disease in the elderly and clinically featured by motor symptoms, such as bradykinesia, muscular rigidity, rest tremor, and postural and gait impairment, and non-motor symptoms, such as olfactory dysfunction, cognitive impairment, psychiatric symptoms, sleep disorders, autonomic dysfunction, pain, and fatigue [42]. The pathological changes of PD are mainly the loss of dopaminergic neurons and other pigment-containing neurons, especially in the substantia nigra pars compacta (SNpc), and the appearance of eosinophilic inclusion bodies - Lewy bodies, in the cytoplasm of residual neurons. Sleep disorders are very common in PD with an incidence of about 60-70% [43], and may have a significant negative impact on the quality of life of these patients [44]. Sleep disturbance in PD includes difficulties in falling or staying asleep, sleep fragmentation and daytime sleepiness with involuntarily daytime naps and RBD [45]. In addition, patients with PD are often affected by primary sleep disorders - especially restless legs syndrome (RLS) or obstructive sleep apnea [44].

Many recent studies have shown that RBD in PD marks a subtype of disease characterized by increased risk of cognitive dysfunction and dementia, more autonomic dysfunction, and more multiple gait dysfunctions [46, 47]. Recent studies have shown that RBD is associated with cholinergic denervation [48]. There may be a common cholinergic mechanism between RBD and subsequent increases in the risk of dementia in PD [49]. A meta-analysis has summarized that the prevalence of RBD in PD is about 42.3% [50], and more than 70% of PD patients were male and suffered from dementia in the RBD group [51]. In the semiquantitative analysis of synaptic nucleoprotein deposition, PD patients with RBD usually exhibit more advanced deposition in each brain region examined [51]. The burden of cerebrovascular disease in patients with PD combined with RBD is slightly higher than that in patients without RBD [51]. PD patients with RBD usually have brain structural network alterations on MRI, showing significant enhanced nodal properties in limbic system, frontal-temporal regions, and occipital regions and decreased nodal properties mainly in cerebellum when compared with PD patients without RBD [52].

Pharmacological treatment is not always deemed necessary in RBD. This requires the joint efforts of the patients and co-sleepers. According to Schenk's pioneering observations, clonazepam is the first line-drug to improve the symptom for the majority of patients [53]. Given the long half-life of the drug, careful monitoring must be performed, especially for patients with advanced disease and a higher risk of falls and cognitive impairment [53]. Melatonin has been proposed as second useful agent with fewer side effects [54]. Pramipexole may also be considered, although some studies yield contradictory results [55]. On the other hand, medications used to treat PD, such as monoamine oxidase inhibitors, have been reported to induce RBD symptoms [56].

### RBD and Parkinsonism plus syndrome

Parkinsonism plus syndrome is a group of neuro-degenerative diseases featuring the classical features of PD (tremor, rigidity, akinesia/bradykinesia, and postural instability) with additional features that distinguish them from PD, including MSA, PSP, and CBD.

MSA is an adult-onset, sporadic neurodegenerative disease with clinical manifestations of combination of parkinsonian features, cerebellar ataxia, autonomic failure, urogenital dysfunction, and corticospinal disorders [57]. The etiology of MSA is still unclear and most of the current studies suggest that the disease may be caused by a disturbance of synucleinopathy [58]. Sleep disorders are common clinical manifestations of MSA, including reduced and fragmented sleep, excessive daytime sleepiness, RBD, and sleep disordered breathing. Among them, RBD is the most common, affecting 68.8-100% of MSA patients [33, 59]. RBD may be the first symptom of MSA, ahead of other clinical manifestations for many years [60, 61]. Sleep disorders occur in both subtypes of MSA, MSA with predominant parkinsonism (MSA-P) and MSA with predominant cerebellar ataxia (MSA-C) [62]. Patients with MSA have a significantly high rate of RSWA [62]. Due to the progressive deterioration of the neuronal structure in the brainstem, RBD symptoms in MSA patients may disappear as their neurological disease worsens. In addition, degeneration of brainstem nuclei that control REM sleep in patients with advanced MSA may mask RBD symptoms [33]. Concerning the treatment of RBD, it is roughly the same as the treatment of RBD in PD, mainly including the safety management of patients and bed partners and drug treatments [4].

PSP is a progressive tauopathy characterized by supranuclear ophthalmoplegia, pseudobulbar palsy, dysarthria, axial rigidity, frontal lobe dysfunction, and dementia [63]. The typical pathology includes neuronal loss, gliosis and MAPT-positive inclusions in neurons and glial cells, primarily in basal ganglia, brainstem and cerebellum [64]. A study find that RBD and RSWA are presented in 13% PSP patients [13]. The severe loss of cholinergic neurons in the pedunculopontine tegmentum may contribute to the absence of normal atonia in REM sleep [13]. RBD can be pharmacologically treated with drugs such as clonazepam or melatonin at night [53]. Recently, a study has reported the beneficial effects of combining the dopamine agonist pramipexole with clonazepam [65]. On the other hand, taking paroxetine, fluoxetine, imipramine, venlafaxine, mirtazapine, and β-blockers can aggravate RBD [53, 62, 66].

CBD is a rare and progressive neurodegenerative disorder. It can present a variety of phenotypes, none of which is specific enough to lead to a definitive diagnosis. One of typical clinical manifestations is corticobasal syndrome, which is usually characterized by asymmetric parkinsonism, a random combination of ideomotor apraxia, rigidity, myoclonus, and dystonia [67]. The ultimate cause of CBD is unclear. However, abnormal accumulations of the MAPT are found in both neurons and glia, supporting the role of tau pathology in CBD [67]. The literatures on RBD in the CBD are mainly limited to case reports and this may be a result of rarity of CBD. 2 female CBD cases were reported with RBD by PSG recording [38, 68]. Degenerative process in cortical and subcortical structures and in the nuclei of the brainstem and pedunculopontine pathways might play a role in the pathogenesis of RBD in CBD [38].

### RBD and Alzheimer’s disease

AD is the most common cause of dementia in the elderly and characterized by the loss of cognitive function and behavioral disorders. The pathological hallmarks of AD are senile plaques deposited by amloid β (Aβ), neurofibrillary tangles (NFTs) formed by abnormal phosphorylated tau (p-tau) and neuronal loss. It is reported that 24.5-40% AD patients have sleep disorders, mainly manifesting as frequent awakenings at night, agitated behavior in the evening and excessive sleeping in the daytime [69]. Sleep disturbance caused by RBD might contribute to Aβ and tau pathologies and aggravate cognitive impairment [70].

Although it is generally considered that RBD is a strong predictor of neurodegeneration in particular synucleinopathies, studies have indicated RBD in a minority of AD patients [12]. A study of probable AD patients has showed that 4 out of 15 patients with AD presented RSWA, and one of these patients had all the polysomnographic features of RBD [71]. Another study of 105 probable AD patients has reported that 5 patients with AD exhibited RBD [12]. AD patients with RBD usually show increases in both tonic and phasic electromyography activity during REM sleep, but no sleep architecture differences between AD patients with and without RBD [12]. A MRI study has showed more specialized atrophic patterns distributed in the posterior and inferior parts of the brain, including the bilateral temporal and occipital cortices in AD patients with RBD compared to non-RBD AD patients [72]. On the other hand, a longitudinal study with about 4.2-year follow-up has demonstrated that 18 out of 84 patients with iRBD developed neurodegenerative diseases, and 3 of these patients were diagnosed with AD [73]. Another study of 179 iRBD patients with mean follow-up of 5.8 years has found that 50 (27.9%) iRBD patients turned out to have neurodegenerative diseases, 14 of these patients were diagnosed with probable AD [74]. Because the neurotransmitter acetylcholine is related to the induction of atonia during REM sleep, it is suggested that the incidence of RBD is associated with decreased acetylcholine level in AD patients [12].

For the treatment of RBD, modifying the sleep environment is recommended to avoid sleep-related injury. Clonazepam is suggested for the treatment of RBD but should be used with caution in patients with dementia, gait disorders, or concomitant obstructive sleep apnea (OSA) [53]. Melatonin has been reported as an alternative treatment in AD patient with RBD and sleep disordered breathing [75]. And medication for the treatment of dementia, such as rivastigmine, might induce RBD in AD patients [76].

### RBD and other dementias

Apart from AD, other diseases with different pathogeneses also cause cognitive impairment and lead to dementia, including DLB, FTD, and vascular dementia (VaD).

DLB is characterized by cognitive impairment, Parkinson’s syndrome and visual hallucinations [77]. It is pathologically featured by intracytoplasmic inclusions called Lewy bodies, which consist of filamentous protein granules composed of α-syn and ubiquitin found in brainstem nuclei and the neocortex [77]. DLB also demonstrates some pathological features of AD, including Aβ deposits and NFTs [77]. An autopsy study has reported that striatal Aβ-immunoreactive plaques are found in DLB and positively correlated with the severity of dementia [78]. Aβ deposition usually predicts DLB cases with high specificity and sensitivity [79]. Another study has found that 71% of the DLB patients are amyloid positive and 17% of the DLB patients are also positive for p-tau [80]. These results imply that AD-like pathologies might play a crucial role in pathogenesis of DLB [78]. Patients with DLB usually have a high prevalence of RBD around 46.7-47.6% [81, 82]. A PSG study has reported that 83% DLB patients showed RSWA with or without dream enactment during PSG [83]. RBD is considered as a strong predictor of neurodegeneration which precede for many years the onset of synucleinopathy [84]. Longitudinal study has reported that RBD might predict the new development of hallucinations and cognitive fluctuations while RSWA might predict development of dementia in PD patients [85]. DLB patients with RBD also exhibits brain metabolic differences, presenting severe metabolic decrease in the dorsolateral and medial frontal regions, left precuneus, bilateral superior parietal lobule and rolandic operculum, and amygdala [80]. A MRI study has reported that the presence of RBD is associated with a higher likelihood of DLB and less severe AD-related pathology in the medial temporal lobes, whereas absence of RBD is characterized by AD-like atrophy patterns on MRI and increased p-tau burden, indicating that RBD is specifically linked to synucleinopathy [82]. Melatonin, clonazepam, and ramelteon can be used to treat RBD in DLB [86].

FTD is characterized by deterioration in behavior and personality, language disturbances, or alterations in muscle or motor functions, resulting from progressive neurodegeneration of the frontal and temporal lobes [87]. The pathogenesis of FTD is heterogeneous due to the different molecular subtypes of clinical FTD [88]. Most frontotemporal degenerations are associated with the MAPT, TAR DNA-binding protein with molecular weight 43 kDa (TDP-43), and fused-in-sarcoma (FUS) protein [87]. A few cases of frontotemporal lobar degeneration have ubiquitin-only or p62-only positive inclusions, or no inclusions at all [87]. It is reported that 76% patients with FTD exhibit sleep disturbance, increased nocturnal activity and decreased morning activity [81]. RBD is rarely reported in FTD and may be misdiagnosed due to the high rate of nocturnal activity and altered sleep-wake cycle in FTD patients [89]. A case report has documented PSG-confirmed RBD in an FTD patient, and the symptoms of RBD include unpleasant dreams, vocalization, waving hands to fight and kicking legs, preceed emotional and behavioral changes of FTD by 3 years [90]. Olanzapine and clonazepam are often prescribed to treat FTD with RBD [90]. Since chromosome 9 open reading frame 72 (C9ORF72) repeat expansion is reported presented in RBD [91], which has also been reported to occur in 25.1 % of FTD patients, C9ORF72 expansion might be responsible for the evolution of RBD in FTD [89].

VaD is a cognitive impairment caused by vascular conditions which reduce the blood flow to the brain. The symptoms of VaD depend on the brain areas affected by the hypoperfusion or hemodynamic abnormality [92]. Previous study has indicated that sleep disturbance, including insomnia, sleep-disordered breathing, RBD, restless legs syndrome, excessive daytime sleepiness occurred in 81.4% of the patients with VaD, and 25.6% VaD patients have RBD [81]. More recent study has reported a higher prevalence (72.6%) of RBD in VaD patients [93]. Since lesions in the LC or SLD cause RSWA, hypoperfusion or hemodynamic abnormalities in these brain areas that regulate REM sleep may contribute to the pathogenesis of RBD in VaD [4].

### RBD and Huntington’s disease

HD is a progressive, neurodegenerative disorder that usually causes movement, cognitive and psychiatric problems with a wide spectrum of signs and symptoms [94]. It is a monogenic autosomal dominant disorder by a CAG triplet repeat expansion in huntingtin (*HTT*) gene, which encodes an expanded polyglutamine stretch in the HTT protein [94]. Evidence suggests that HD arises predominantly from gain of a toxic function from an abnormal conformation of mutant HTT [94]. Sleep disorders in HD include frequent insomnia, earlier sleep onset, lower sleep efficiency, increased stage 1 sleep, delayed and shortened REM sleep, and increased periodic leg movements [14]. Circadian rhythm disruption is also a prominent feature of sleep disorders in HD [95]. A study has reported that 3 out of 25 HD patients have RBD, manifesting as complex movements of the lips, head, trunk, and right hand and arm during REM sleep [14]. However, in another polysomnographic study, no episode of RBD is observed in 30 HD patients [96].

### RBD and amyotrophic lateral sclerosis

ALS is an adult-onset progressive neurodegenerative disease, characterized by rapidly progressive loss of cortex, spinal cord, bulbar movement neurons, manifesting with paralysis of the striatum skeletal muscle, bulbar muscle, dysphagia, dysarthria, and respiratory dysfunction [97]. ALS patients usually die in 1-5 years resulting from respiratory failure [97]. ALS patients often suffer from sleep disorders including insomnia, fragmented sleep, and increased periodic limb movement disorder [15]. Previous studies have focused on sleep disorders caused by chronic respiratory muscle paralysis and hypoventilation in ALS patients [98, 99]. RBD has also been reported in ALS patients. A study of 41 ALS patients and 26 healthy participants has found that 2 ALS patients had RBD, 2 had RSWA, and the control group had normal REM sleep [15]. Mechanism of RBD in ALS patients is uncertain. The early death caused by respiration in ALS patients affects the observation of RBD in later stage [97]. Moreover, the absence of ALS motor neurons also prevents the motor behaviors during sleep. During REM, the tone of the chin muscles changed in ALS [97]. However, it is uncertain if the alteration of the muscle tone during REM sleep results from the loss of innervation because of the degeneration of lower motor neurons, or if it can parallel spasticity and hyperreflexia associated with the degeneration of upper motor neurons [97]. The most common forms of ALS and FTD are caused by GGGGCC hexanucleotide repeat expansions in the first intron of C9ORF72 [100]. A study of 344 patients with RBD has found that 2 RBD patients have the same C9ORF72 associated-risk haplotype identified in ALS and FTD, suggesting that this rare expansion might be associated with the pathogenesis of RBD [91].

**Table 1:** Summarized prevalence, gender difference and underlying mechanisms of RBD in neurodegenerative diseases.

Disease	Prevalence	Gender difference	Possible mechanisms of pathogenesis of RBD
PD	42.3%[50]	Male>female [51]	α-syn pathology affects the circuit that regulates REM sleep, associated with *GBA* [39], *SCARB2* [40], *MAPT* [40], *USP25*[40], *PINK1* [41], *LRRK2* [2] mutations
MSA	88%[33]	Female>male [59]	α-syn pathology affects the circuit that regulates REM sleep, depletion of cholinergic neurons in the PPN/LDTN complex, periaqueductal grey matter, and LC [33]
PSP	13%[13]	-	Loss of cholinergic neurons in the pedunculopontine tegmentum [13]
CBD	Case reports[38, 68]	2 female patients [38, 68]	Degenerative process in cortical and subcortical structures and in the nuclei of the brain stem and pedunculopontine pathways [38].
AD	4.8-26.7%[12, 71]	Male>female [12]	An imbalance of neurotransmitter acetylcholine [12], neuronal loss in LC [12]
DLB	46.7-83%[81-83]	Male>female [82]	α-syn pathology affects the circuit that regulates REM sleep [4]
FTD	Rare (only case report)[90]	1 male patient [90]	Associated with C9ORF72 repeat expansion [89]
VaD	25.6%-72.6%[81, 93]	-	Hypoperfusion or hemodynamic abnormalities affect the brain areas that regulate REM sleep [4]
HD	12% or lower[14, 96]	Female>male [14]	Associated with mutant huntingtin [14]
ALS	4.9%[15]	2 male patients [15]	Neurodegeneration of nuclei in REM-associated pathways in the brainstem and the dysfunction of dopaminergic system in substantia nigra striatum may be the main pathophysiological culprit in the development of RBD [15], associated with C9ORF72 repeat expansion [91, 100]
CJD	7.1%[101]	2 male patients [101]	Associated with corticothalamic degeneration [102]

Abbreviations: AD, Alzheimer’s disease; ALS, amyotrophic lateral sclerosis; CBD, corticobasal degeneration; CJD, Creutzfeldt-Jakob disease; DLB, dementia with Lewy bodies; FTD, frontotemporal dementia; GBA, glucose encephalo-glucosidase; HD, Huntington’s disease; LC, locus coeruleus; LDTN, laterodorsal tegmental nucleus; LRRK2, leucine rich repeat kinase2; MAPT, microtubule associated protein tau; MSA, multiple system atrophy; PD, Parkinson’s disease; PINK1, PTEN-induced putative kinase 1; PPN pedunculopontine tegmental nucleus; PSP progressive supranuclear palsy; RBD, rapid eye movement sleep behavior disorder; REM, rapid eye movement; SCARB2, scavenger receptor class B member 2; USP25, ubiquitin specific peptidase 25; VaD, vascular dementia.

### RBD and Creutzfeldt-Jakob disease

Creutzfeldt-Jakob disease (CJD) is a rapid progressive, fatal neurodegenerative diseases. The main pathogenesis is the accumulation of misfolded prion protein, PrP^Sc^ [101]. Sleep disorders are common in CJD. Early treatment and prevention of sleep disorders can interfere with the treatment of CJD. Although sleep symptoms are not considered to be the main symptoms of CJD, sleep-wake disorder is an important source of morbidity and reduces the quality of life in these patients [101]. A cross-sectional cohort study has retrospectively analyzed 28 patients diagnosed CJD and found that 3 patients had RSWA, and 2 had RBD [101]. This study suggests that RBD is as common in CJD patients as it is in patients with synucleinopathies. RBD in CJD might result from corticothalamic degeneration [102]. Due to methodology and limited sample size, larger studies involving multiple centers are needed.

### Summary

RBD is generally considered to be associated with synucleinopathy, but it is also reported in other neurodegenerative diseases including AD, FTD, HD, ALS, etc, which are not characterized by α-syn pathology. The summarized prevalence, gender difference and underlying mechanisms of RBD in neurodegenerative diseases is listed in Table 1. RBD may precede years before the onset of the symptoms of synucleinopathy and predict neurodegeneration and cognitive impairment. The underlying mechanisms of the pathogenesis of RBD is not clear. Since the neural circuit in caudal brainstem that regulates REM sleep is where α-syn pathology may begin in synucleinopathy, RBD may result from the degeneration of circuits that regulate the REM sleep, suggesting that the α-syn pathological process may start years before the motor or cognitive symptoms of these diseases. Sleep changes might be a very early sign of neurodegeneration in synucleinopathy. On the other hand, RBD in PD is associated with a more impaired cognitive profile, suggesting more severe and widespread neurodegeneration [103], implying sleep disturbance might also be an assessment of cognitive deficits in synucleinopathies. In non-synucleinopahties, RBD may be associated with co-existing α-syn pathology [104] or other pathological changes affecting the pathways in the brainstem controlling REM [13, 15, 38]. Thus, the site of the lesion is more critical in the pathogenesis of RBD, rather than the type of pathological changes. For the treatment of RBD, clonazepam, melatonin, and ramelteon can be applied. Medications used to treat the neurodegenerative diseases, such as monoamine oxidase inhibitors, antidepressant, might induce or deteriorate the symptoms of RBD.
